# Supplementary figures and images for: Proteomic Characterization of the Olfactory Molecular Imbalance in Dementia with Lewy Bodies
Source: Int J Mol Sci. 2020 Sep 2;21(17):6371. doi: 10.3390/ijms21176371 (PMC7503830; doi:10.3390/ijms21176371)

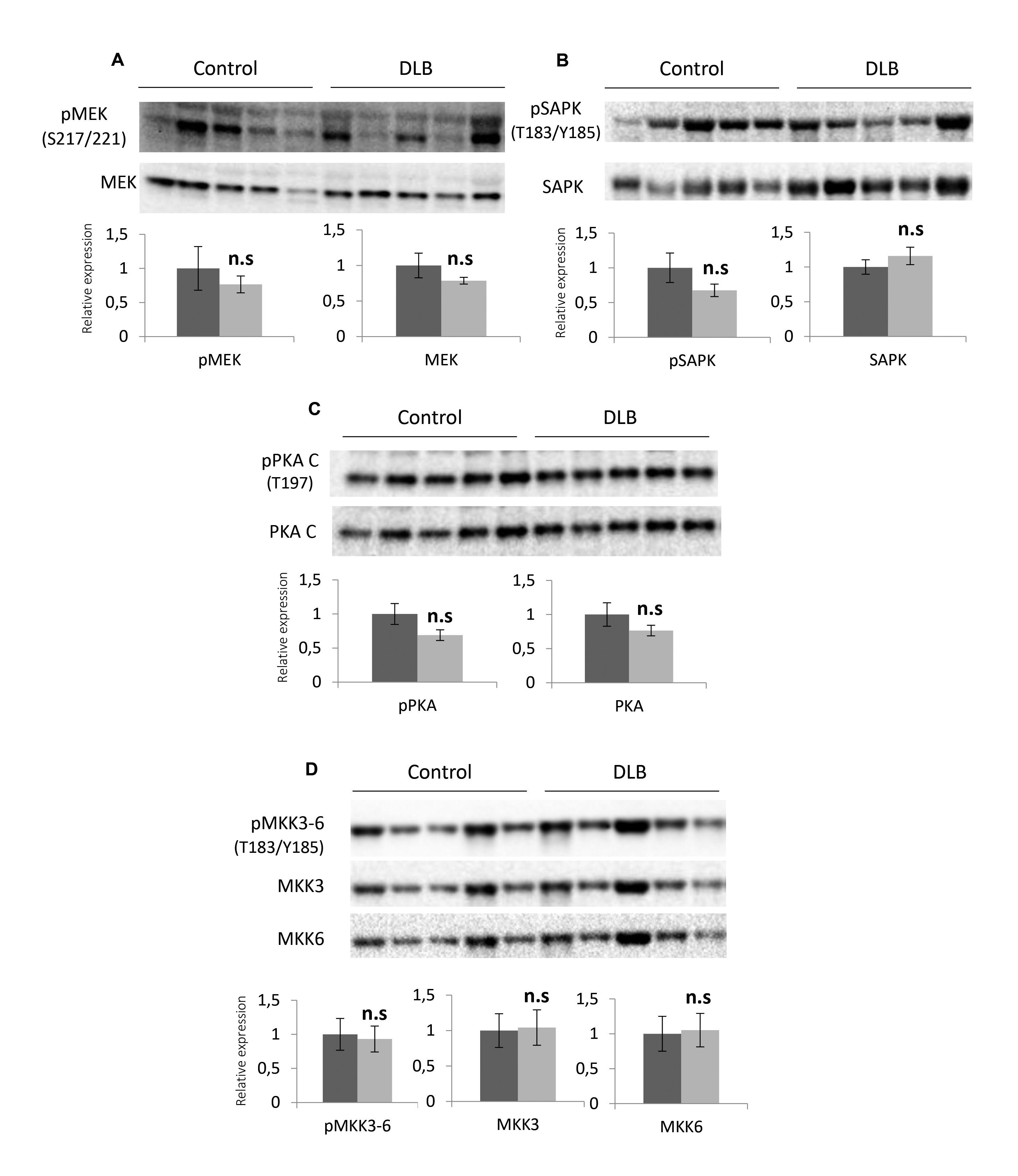

Supplement: Supplementary file 1 [file ijms-21-06371-s001.zip › Supplementary Figure S1.tif]

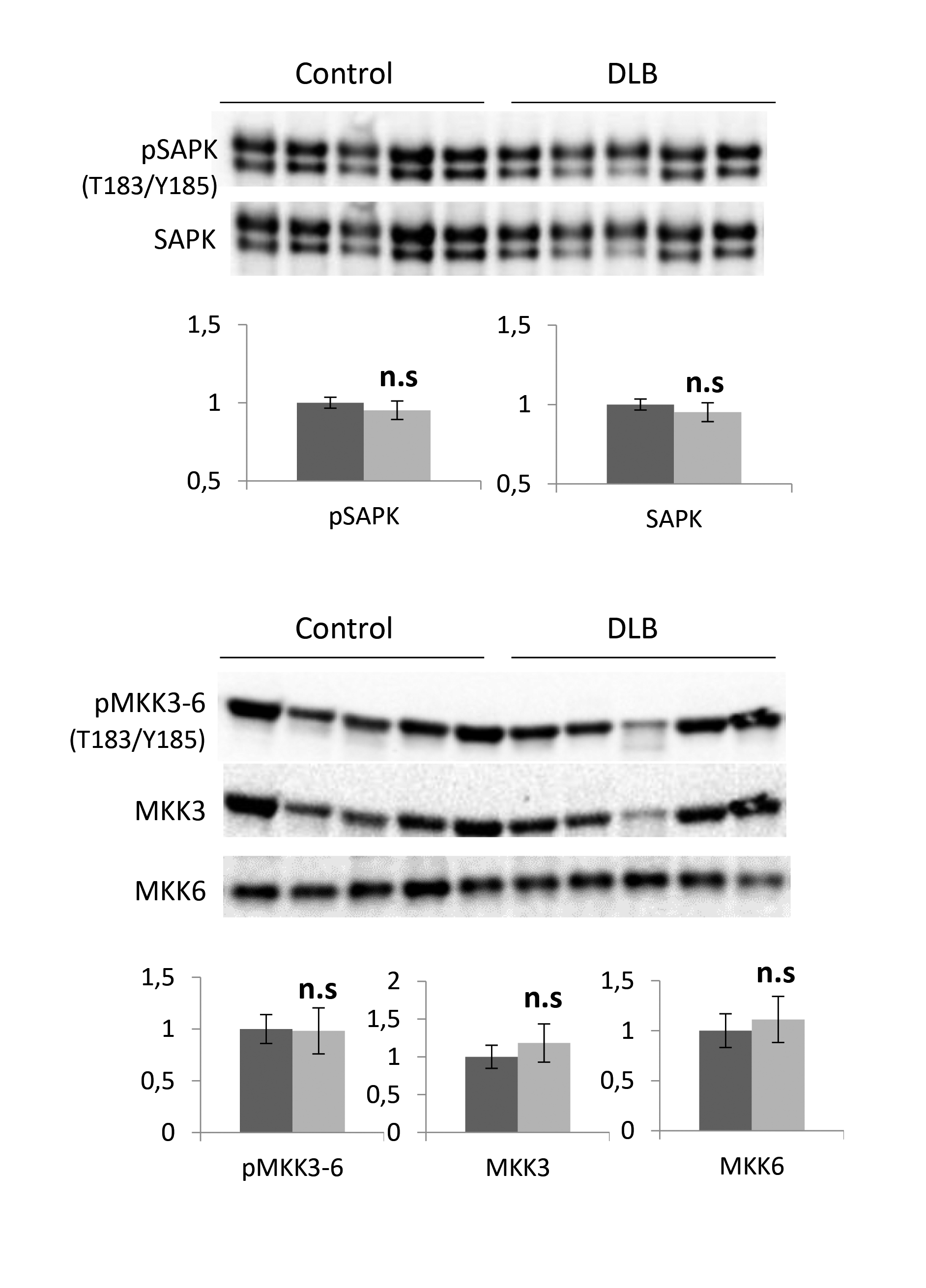

Supplement: Supplementary file 1 [file ijms-21-06371-s001.zip › Supplementary Figure S2.tif]

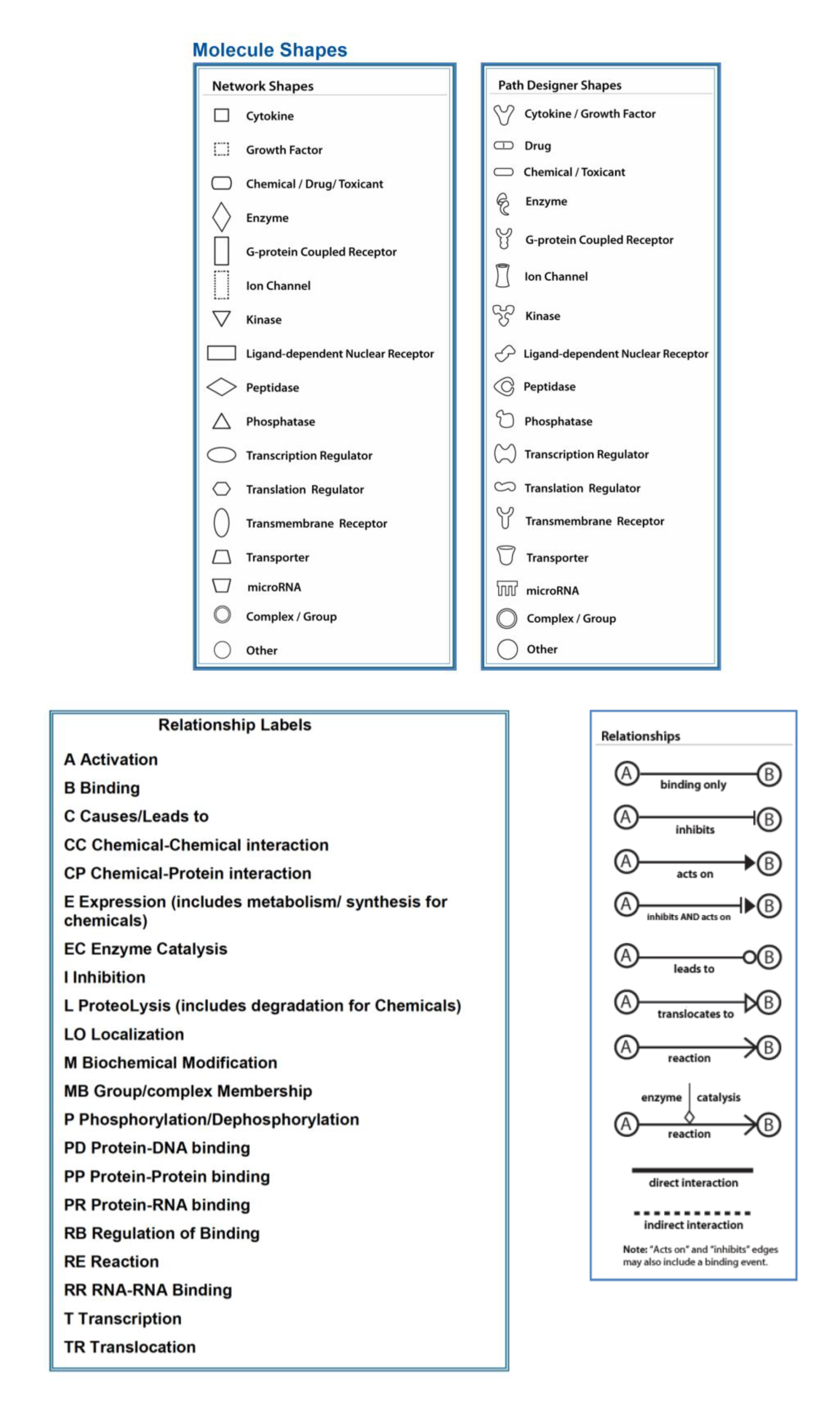

Supplement: Supplementary file 1 [file ijms-21-06371-s001.zip › Supplementary Figure S3.tif]

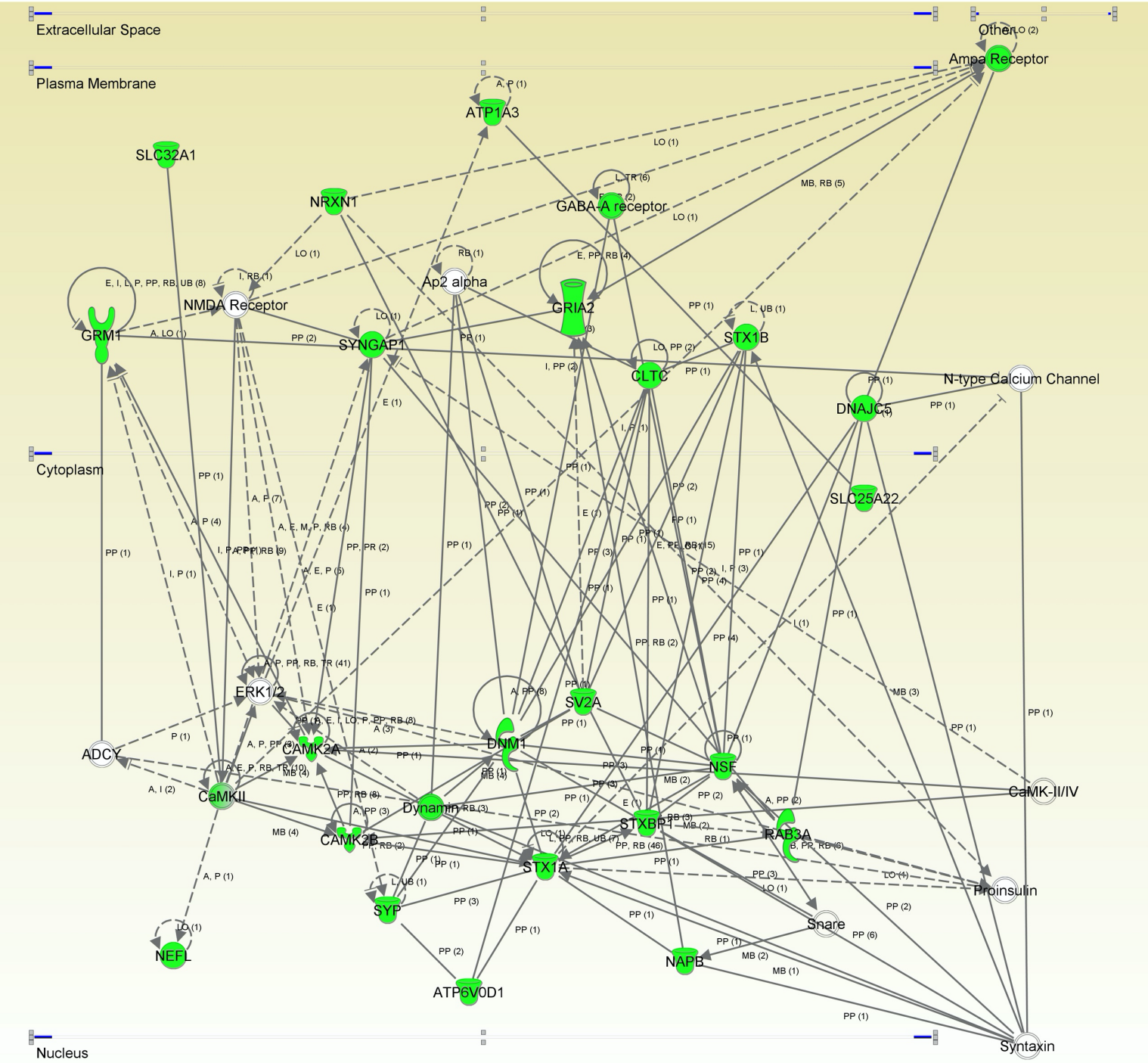

-Supplementary figure 5-

Supplement: Supplementary file 1 [file ijms-21-06371-s001.zip › Supplementary Figures S4 and S5.pdf]
